# Acute Drug Treatment in the Early *C. elegans* Embryo

**DOI:** 10.1371/journal.pone.0024656

**Published:** 2011-09-14

**Authors:** Ana Carvalho, Sara K. Olson, Edgar Gutierrez, Kelly Zhang, Lisa B. Noble, Esther Zanin, Arshad Desai, Alex Groisman, Karen Oegema

**Affiliations:** 1 Ludwig Institute for Cancer Research, Department of Cellular and Molecular Medicine, University of California San Diego, La Jolla, California, United States of America; 2 Department of Physics, University of California San Diego, La Jolla, California, United States of America; Centre for Genomic Regulation, Spain

## Abstract

Genetic and genome-wide RNAi approaches available in *C. elegans*, combined with tools for visualizing subcellular events with high-resolution, have led to increasing adoption of the early *C. elegans* embryo as a model for mechanistic and functional genomic analysis of cellular processes. However, a limitation of this system has been the impermeability of the embryo eggshell, which has prevented the routine use of small molecule inhibitors. Here, we present a method to permeabilize and immobilize embryos for acute inhibitor treatment in conjunction with live imaging. To identify a means to permeabilize the eggshell, we used a dye uptake assay to screen a set of 310 candidate genes defined by a combination of bioinformatic criteria. This screen identified 20 genes whose inhibition resulted in >75% eggshell permeability, and 3 that permeabilized embryos with minimal deleterious effects on embryo production and early embryonic development. To mount permeabilized embryos for acute drug addition in conjunction with live imaging, we combined optimized inhibition of one of these genes with the use of a microfabricated chamber that we designed. We demonstrate that these two developments enable the temporally controlled introduction of inhibitors for mechanistic studies. This method should also open new avenues of investigation by allowing profiling and specificity-testing of inhibitors through comparison with genome-wide phenotypic datasets.

## Introduction

Small molecule inhibitors are a valuable tool for the analysis of fundamental cellular functions and an entry point for the development of therapeutic agents. In mechanistic studies, the primary advantage of small molecules is temporal control, which is especially powerful when combined with live imaging. Drug delivery experiments are straightforward in tissue culture cells, where drugs can simply be added to the medium. However, in *C. elegans* embryos, where RNA-interference (RNAi)-based analysis has generated extensive phenotypic datasets and provided fundamental insights into many cellular processes, use of small molecule inhibitors has been limited due to eggshell impermeability.

Laser puncturing [1] or pressure against an overlying coverslip [2] have been used to permeabilize the *C. elegans* eggshell for drug delivery, but both techniques have significant drawbacks that preclude their routine use. Laser puncturing requires a sophisticated setup and confines drug entry to a single narrow opening, which leads to slow and spatially non-uniform introduction of inhibitors into the embryo. The compression method is prone to disrupt embryonic processes and is difficult to control. Eggshells have also been weakened in bleach followed by chitinase treatment and passing the embryos through a narrow mouth pipette [3]; however, this method often ruptures embryos and is difficult to execute consistently.

Here, we present a simple method for generating uniformly permeable *C. elegans* embryos and immobilizing them to allow acute drug treatment with simultaneous live imaging. To identify a means to permeabilize embryos, we performed an RNAi-based screen of a set of 310 candidate genes. This screen identified 3 genes whose inhibition yielded penetrant embryo permeability with minimal deleterious effects on embryo production and early embryonic development. To mount permeabilized embryos for acute drug addition in conjunction with live imaging, we combined optimized inhibition of one of these genes with the use of a microfabricated chamber that we designed. We demonstrate the efficacy of this method by treating embryos with three different small molecule inhibitors.

## Results and Discussion

**Figure 1:**
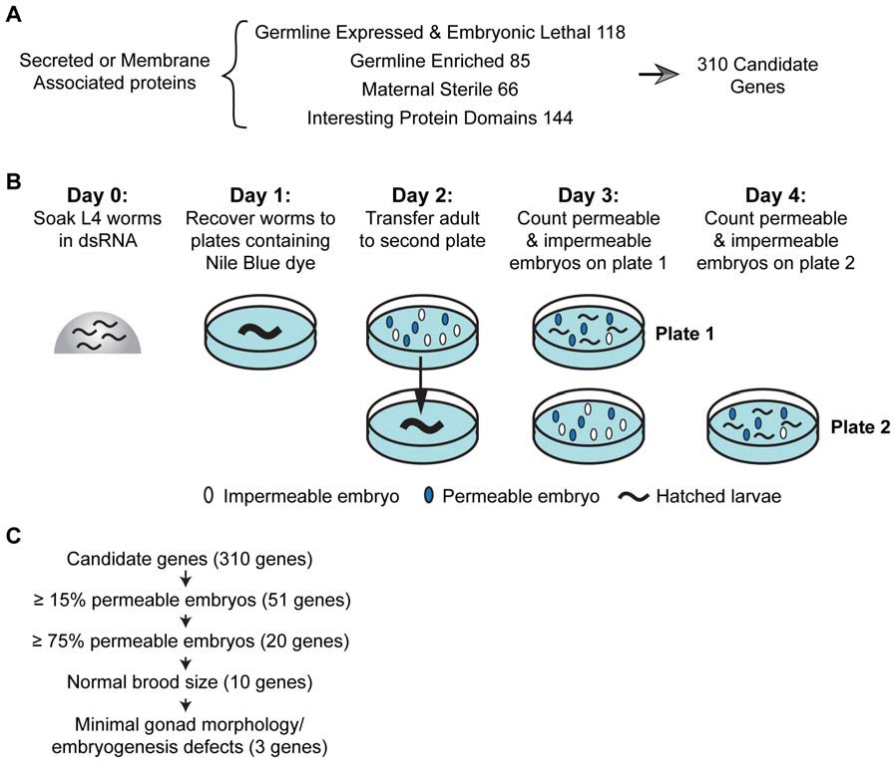
A screen for genes required for eggshell impermeability. **A.** Since the eggshell is a secreted structure, all candidate genes encoded proteins with a signal peptide or a membrane-targeting domain. Our candidate gene set included all of the germline enriched ([13], [14]) and maternal sterile ([5]) genes that have a signal peptide. Since the set of genes expressed in dissected germlines that have a signal peptide is large (1137 genes, Serial Analysis of Gene Expression database, Genome BC *C. elegans* Gene Expression Consortium, http://elegans.bcgsc.bc.ca) we included only those previously shown to result in embryonic lethality when inhibited by RNAi (Wormbase release WS199). Genes with proteins domains (chitin binding, glycosylation, protease, peroxidase, lipid binding, or LDLR domains) found in proteins implicated in eggshell formation or analogous processes in other species were included regardless of whether there was prior evidence for a germline role. Due to redundancy, the total number of candidate genes was 310. **B.** Outline of screen to identify genes specifically required for eggshell impermeability. **C.** Of the 310 candidate genes tested, 3 genes were identified whose inhibition permeabilized the eggshell with high penetrance (>75% permeable embryos), while maintaining a normal broodsize and leading to minimal defects in gonad structure/embryonic development. One of these genes, *perm-1*, was chosen for further optimization.

**Figure 2:**
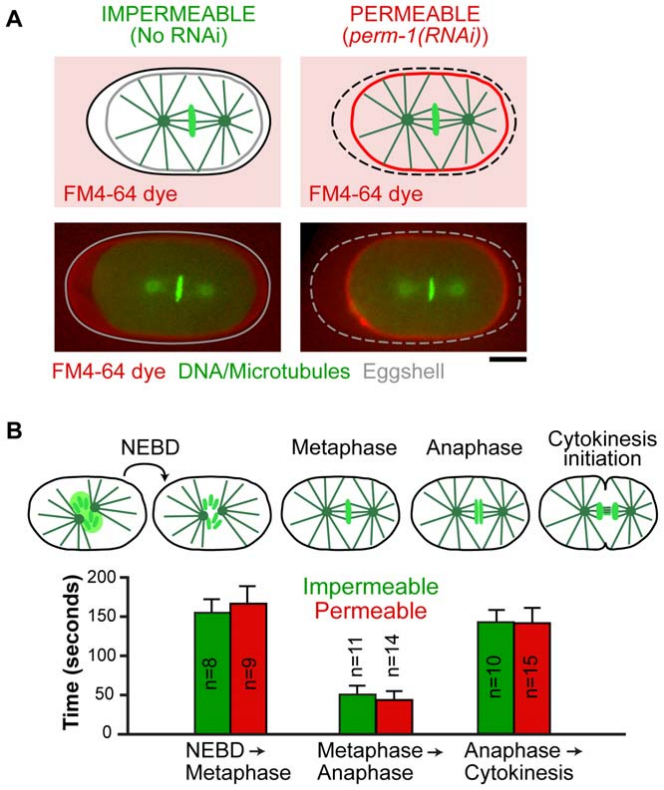
*perm-1* inhibition permeabilizes the eggshell without altering cell division timing. **A.** Schematics and confocal images of impermeable (no RNAi) and permeable (*perm-1(RNAi)*) embryos co-expressing GFP-histone H2B and GFP-alpha-tubulin. Embryos at metaphase of the first mitotic division were submerged in media containing the lipophilic dye FM4-64, which passes through the eggshell to label the plasma membrane of permeable (***right***), but not impermeable (***left***) embryos. Scale bar, 10 µm. **B.** Time intervals between NEBD and metaphase, metaphase and anaphase, and anaphase and cytokinesis onset were measured for permeable embryos (*perm-1(RNAi)*); ***red bars***) and impermeable wild-type controls (***green bars***).

To identify an RNAi-based means to permeabilize the *C. elegans* eggshell to allow introduction of small molecule inhibitors while maintaining normal progression through the early embryonic cell divisions, we screened through a collection of 310 candidate genes, chosen based on their primary sequence features and/or phenotypic profiles (Fig. 1A; see Spreadsheet S1.xlsx for candidate gene selection details). Worms were soaked in dsRNA targeting each candidate gene and placed onto plates containing the dye Nile Blue A; dye uptake into the embryos indicates eggshell permeability (Fig. 1B) [4]. This screen identified 51 genes whose inhibition resulted in >15% permeable embryos and 20 that resulted in >75% embryo permeability. Penetrant (>75%) embryo permeability combined with a normal brood size was observed for 10 genes (Fig. 1C; Table 1; for a complete screen summary see Spreadsheet S1.xlsx); four of these genes were previously uncharacterized, and we named them *dgtr-1* (DGAT-related), *egg-6 (EGG sterile)*, *perm-1* and *perm-2* (for PERMeable eggshell). Sequence comparisons suggested that *dgtr-1* is related to the DGAT/MGAT family of enzymes and is likely to be involved in lipid synthesis. Of these 10 genes, inhibition of 7 had previously been shown to lead to defects in gonad structure [5] or to disruption of events in the early embryo [6], [7], [8], [9], [10]. Inhibition of the remaining three genes (*dgtr-1*, *perm-1*, and *ptr-2*) resulted in minimal deleterious effects on embryo production and on early embryonic development. We selected *perm-1* (T01H3.4), for additional optimization. Partial inhibition of *perm-1* gave rise to embryos permeable to the lipophilic dye FM4-64 (Fig. 2A) that progressed through the early embryonic cell divisions with normal timing (Fig. 2B). Embryos permeabilized by *perm-1* inhibition did exhibit late embryonic lethality; however, this has also been observed following permeabilization via other means [3], [11], [12] and may reflect a requirement for an impermeable eggshell to properly execute morphogenetic events during later embryonic development. Thus, as with other permeabilization methods, *perm-1(RNAi)* allows access to events during the early embryonic cell divisions.

**Table 1:** Screen hits (>75% permeable embryos & brood size* >90).

Target ORF	Gene name	Gene description	NCBI KOG	Human homolog
***Lipid biosynthesis, modification, or sensing***				
F32H2.5	*fasn-1*	Fatty Acid SyNthase	None	FASN (NP_004095.4)
H02I12.8/Y17G9B.3	*cyp-31A2/3*	CYtochrome P450 family	Cytochrome P450 CYP4/CYP19/CYP26 subfamilies	CYP4V2 (NP_997235.3)
K10D2.6	*emb-8*	Cytochrome P450 NADPH-reductase	NADP/FAD dependent oxidoreductase	POR (NP_000932.3)
W01A11.2	*dgtr-1*	(DGAT-Related); putative Acyl-CoA:diacylglycerol acyltransferase	Acyl-CoA:diacylglycerol acyltransferase	Related to enzymes in the DGAT/MGAT family
C32E8.8	*ptr-2*	PaTched Related	Patched superfamily	PTCHD family
***Glycosylation, chitin-binding***				
C07G2.1/B0280.5	*cpg-1/cpg-2*	Chondroitin ProteoGlycan	None	None
***Meiosis/Mitosis***				
T05G5.7	*rmd-1*	Regulator of Microtubule Dynamics	Uncharacterized conserved protein	Unclear
***Uncharacterized***				
T01H3.4	*perm-1*	PERMeable eggshell	None	None
K07A12.2	*egg-6*	EGG sterile	Leucine rich repeat	None
C44B12.1	*perm-2*	PERMeable eggshell	None	None

*Brood size = total number of hatched larvae, permeable embryos, and dead embryos on the Day1 and Day2 plates combined.

*perm-1(RNAi)* can be performed by soaking, feeding or injection. For all delivery methods, we were able to optimize RNAi conditions to obtain ∼80–100% permeable embryos with no discernable defects during the first two embryonic divisions (Figs. S1, S2). The major defect encountered when *perm-1(RNAi)* conditions are not properly titrated is resorption of the second polar body. Soaking and feeding are the easiest delivery methods, but we have found injection to be the most effective when combining *perm-1(RNAi)* with RNAi of other targets (*perm-1* dsRNA is effective when mixed with a second dsRNA at a ratio of 1 to 6; data not shown).

**Figure 3:**
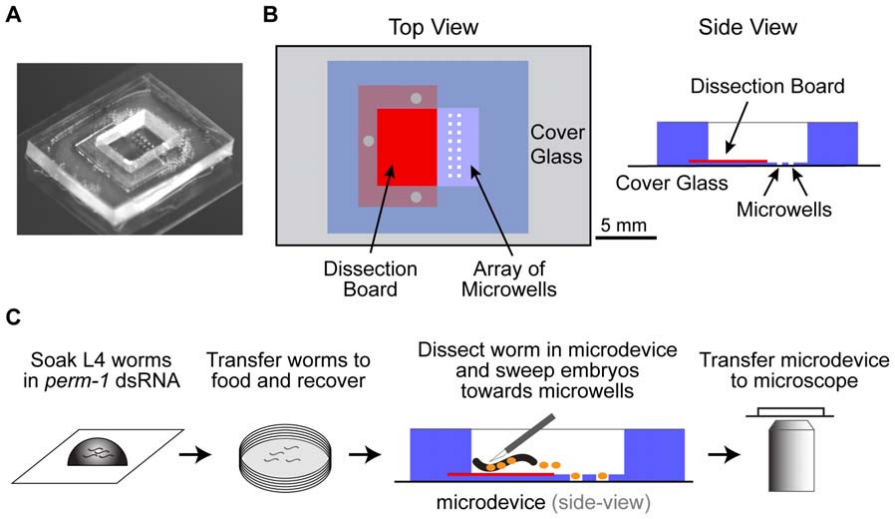
A microdevice that immobilizes permeable embryos for simultaneous imaging and small molecule addition. **A.** Photograph of the microdevice. **B.** Schematic drawings of the top and side views of the microdevice. **C.** Schematic depicting the procedure for preparing permeabilized embryos for drug treatment and imaging. *C. elegans* hermaphrodites at the L4 larval stage are soaked in a drop of *perm-1* dsRNA for 4 hours at 20°C. The worms are transferred to a plate with bacteria and allowed to recover for 16 hours at 16°C. 1–3 worms are placed on the dissection board of the microdevice after filling it with ∼100 µl of media. Worms are dissected with a scalpel and the released early embryos are swept into the microwells with an eyelash tool. The microdevice is transferred to the microscope and the embryos are imaged. When the desired stage is reached, the medium in the microdevice is replaced with fresh medium containing the drug of interest.

We next developed a method to combine *perm-1(RNAi)*-mediated permeabilization with live imaging in a manner that facilitates small molecule addition. For this purpose, the permeabilized embryos needed to be stably mounted during medium exchange. Permeable embryos are more fragile than embryos with an intact eggshell and cannot be compressed under a coverslip or transferred using mouth pipettes. We therefore designed a microdevice that allows the *in-situ* dissection of adult hermaphrodites and immobilization of the released permeable embryos for imaging before and after small molecule addition (Fig. 3). The device consists of a microfabricated composite chip, which is made of silicone elastomer and epoxy, and a #1.5, 24×60 mm coverslip, which is attached to the bottom of the chip. The chip has a rectangular well that has an elevated hard epoxy dissection board on the bottom on one side and an 8×6.4 mm array of 16 microwells on the other (Fig. 3A,B and S3). In the beginning of an experiment, the well and the microwells are filled with osmotic support medium and a worm is placed on the dissection board. The worm is cut open with a scalpel, and an eyelash tool is used to move the released permeable embryos into the microwells, where they rest on the coverslip surface facilitating imaging with an inverted microscope (Fig. 3C). At the desired time of drug addition, the medium in the well is replaced with medium containing the drug of interest. A single cycle of aspiration/dispensing leads to an equilibrium small molecule concentration in the well that is ∼60% of that in the added buffer. Equilibrium with the buffer in the microwells is achieved within ∼5 seconds and the embryos remain motionless during the entire process of buffer exchange (Fig. S3B).

**Figure 4:**
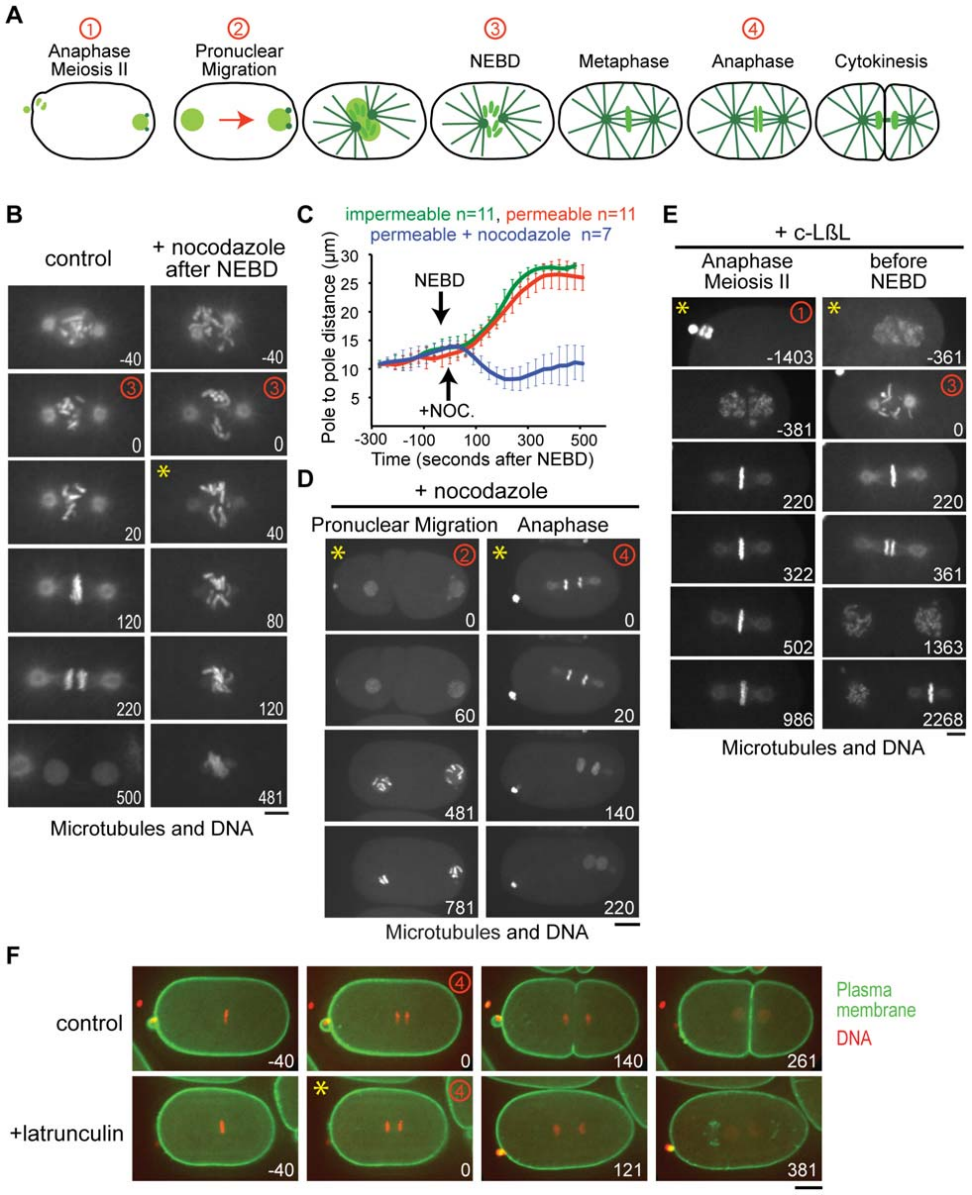
Acute drug treatments with nocodazole, latrunculin A, and c-lactocystin-ß-lactone. **A.** Schematics illustrating the stages between anaphase meiosis II and cytokinesis of the first mitotic division following fertilization. Red numbers mark the timepoints highlighted in the panels in B, D, E and F. **B.** Confocal images of permeable embryos expressing GFP-histone H2B and GFP-alpha-tubulin. A control embryo (***left***) and an embryo treated with 10 µg/ml nocodazole after NEBD (***right***) are shown. Addition of nocodazole results in rapid microtubule depolymerization. Numbers in white indicate time in seconds after NEBD. Scale bar, 5 µm. **C.** Mean pole-to-pole distance plotted versus time for the indicated conditions. Embryos treated with nocodazole after NEBD exhibit a rapid reduction in pole-to-pole distance due to microtubule depolymerization. Error bars are the 95% C.I. of the mean. **D.** Images of permeable embryos expressing GFP-histone and GFP-alpha-tubulin. Nocodazole was added during pronuclear migration (***left***) or in early anaphase (***right***). In both cases, rapid microtubule depolymerization was observed. Scale bar, 10 µm. Numbers in white indicate time in seconds after the timepoint when drug was added. **E.** Images of permeable embryos expressing GFP-histone H2B and GFP-alpha-tubulin. The proteasome inhibitor, c-lactocystin-ß-lactone (20 µM) was added either early (anaphase of meiosis II; ***left***) or just before NEBD (***right***). If added early, the embryo arrested at the first metaphase. If added before NEBD, the metaphase-anaphase transition was delayed, but the embryo did not arrest until metaphase of the second division. Scale bar, 5 µm. Numbers in white indicate time in seconds after NEBD of the first mitotic division. **F.** Images of embryos expressing a GFP-labeled plasma membrane probe and RFP^mCherry^-histone H2B. Latrunculin A (10 µM) added after anaphase onset prevented cleavage furrow ingression. Scale bar, 10 µm. Numbers in white indicate time in seconds after anaphase onset. Yellow asterisks in the upper left corner of the panels in B, D, E and F mark the time point when drug was added.

To confirm that combining *perm-1(RNAi)* with use of the microdevice enables routine small molecule-based experiments in *C. elegans* embryos, we performed treatments with three different inhibitors: the microtubule depolymerizing agent nocodazole, the actin inhibitor latrunculin A, and the proteasome inhibitor c-lactocystin-ß-lactone. All 3 inhibitors had the expected effects: nocodazole caused rapid spindle collapse if added after nuclear envelope breakdown (NEBD; Fig. 4B,C; yellow asterisk marks frame when drug was added, Movie S1). If nocodazole was added earlier, it prevented pronuclear migration; if added just after anaphase onset, it inhibited cytokinesis (Fig. 4D, Movies S2 and S3). Addition of latrunculin A before furrow ingression blocked cytokinesis but not nuclear division (Fig. 4F, Movie S4). The proteasome inhibitor, c-lactocystin-ß-lactone, caused a metaphase arrest when added early (meiosis II, ∼25 minutes prior to metaphase, Movie S5); if added just before mitosis (at NEBD), it delayed the metaphase-anaphase transition but did not cause a metaphase arrest until the subsequent cell cycle (Fig. 4E, Movie S6). Addition of the same inhibitors had no effects on control impermeable embryos. These results establish that *perm-1(RNAi)* and the microdevice can be used to combine precisely controlled inhibitor treatments with dynamic imaging of the early *C. elegans* embryo.

We describe a robust method that overcomes the impermeability of *C. elegans* embryos to allow small molecule inhibitor studies in this widely utilized experimental model. This method enables precisely timed inhibitions, which will greatly enhance ongoing studies of cell division, cell polarity, nuclear membrane assembly/disassembly, membrane trafficking, and cortical dynamics. Combining our method with mutants/RNAi will provide a means for testing inhibitor specificity in an *in vivo* context. We anticipate that the precise phenotypic profiling possible in the early *C. elegans* embryo will also aid in target identification for inhibitors discovered in cell-based screens, given the richness of functional genomic datasets available in this system. Although the microdevice we describe here is optimized for treatment of embryos from a single strain with a single inhibitor, we anticipate that devices with similar designs could allow more high-throughput screening of multiple strains and/or inhibitors.

## Materials and Methods

### Selection of candidate genes for eggshell permeability screen

All candidate genes encode secreted or membrane-associated proteins as judged by the presence of a signal peptide or a predicted transmembrane or signal anchor domain (SignalP 3.0, www.cbs.dtu.dk/services/SignalP; PSORT II, psort.hgc.jp/form2.html) and were selected from a curated list of *C. elegans* secreted proteins provided by Brian Ackley and Andrew Chisholm. For the majority of candidates, we also required at least one line of prior evidence for a role in the germline. Our candidate gene set included all of the germline enriched [13], [14] and maternal sterile genes [5] that had a signal peptide. Since the set of genes previously found to be expressed in dissected germlines that have a signal peptide is large (Serial Analysis of Gene Expression database, Genome BC *C. elegans* Gene Expression Consortium, http://elegans.bcgsc.bc.ca), germline expressed genes were only included if they had previously been shown to result in embryonic lethality when inhibited by RNAi (Wormbase release WS199). Genes with domains found in proteins previously implicated in the formation of extra-embryonic layers (chitin binding, glycosylation, protease, peroxidase, lipid binding, or LDLR domains; Wormbase release WS202) were included regardless of whether there was prior evidence for a germline role. Due to redundancy, the total number of candidate genes was 310 (see Spreadsheet S1.xlsx for a breakdown of genes by identification method).

### Eggshell permeability screen

Oligo design, PCR, RNA transcription and annealing, and soaking RNAi were performed as described in Ref. [5]. The primer pairs used to amplify 500–1000 bp target regions of candidate genes are listed in Spreadsheet S1. For RNAi experiments, five to seven L4 stage larval worms were soaked in dsRNA (or soaking buffer for control experiments) for 24 hr at 20°C and recovered individually to NGM plates containing 150 µg/ml Nile Blue A, which penetrates permeable, but not intact, eggshells [4]. dsRNA-treated adults were allowed to lay embryos for 24 hr at 25°C (Plate 1), then transferred to a fresh plate to lay embryos for an additional 24 hr (Plate 2). One day after the adult was removed, the number of hatched larvae, impermeable embryos (clear), and permeable embryos (blue) were counted for each plate, the sum of which was recorded as brood size. Averages for the 5–7 worms are reported (Spreadsheet S1.xlsx). A maximum of 50 hatched larvae per plate were counted. Control worm plates normally contained 0–4% permeable embryos.

### Soaking or Feeding RNAi, transfer to chamber, addition of small molecules

To generate the permeable embryos in Figs. 2 and 4, dsRNA against T01H3.4 was prepared by using the oligos in parentheses (taatacgactcactataggAATTTTCTAGGTCGTCAATCTTCA, aattaaccctcactaaaggCGAAAACGCGATCATTTTTA) to amplify the corresponding region from N2 genomic DNA. The PCR reaction was cleaned (Qiagen, Valencia, CA) and used as template for 50 µl T3 and T7 transcription reactions (Ambion, Austin, TX). The two reactions were cleaned (MEGAclear kit, Ambion, Applied Biosystems), eluted with 50 µl of DEPC-treated H_2_O each, combined and mixed with 50 µl of 3× soaking buffer (32.7 mM Na_2_HPO_4_, 16.5 mM KH_2_PO_4_, 6.3 mM NaCl, 14.1 mM NH_4_Cl). The RNA was annealed by incubating the mix at 68°C for 10 minutes followed by 37°C for 30 minutes and then frozen down in aliquots. RNAi was performed by placing 20–25 L4-stage worms (strains listed in Table S1) in a drop of 5 µl of dsRNA, after adding 0.25 µl 63 mM spermidine and 0.25 µl 1.1% gelatin, on parafilm in a humid chamber at 20°C. After 4 hours, worms were transferred to a seeded NGM (nematode growth medium) plate and incubated for 16–22 hours at 16°C.

For feeding RNAi, the T01H3.4 clone from the Ahringer library [15] was used. Bacteria expressing dsRNA targeting T01H3.4 were grown overnight at 37°C in LB with 100 µg/ml ampicillin. The overnight culture was diluted 1∶50 in LB with 100 µg/ml ampicillin and grown at 37°C until culture reached an OD_600_ between 0.4–0.7 (∼2.5 hours). The bacterial culture (∼200 µl/plate) was spread onto 6 cm NGM agar plates containing 0.01 mM IPTG. Plates were dried in a sterile hood for ∼1 hour and left at room temperature for ∼4 hours to induce RNA expression. 30–50 L4 or young adult stage worms were transferred onto each plate and incubated overnight at 20°C for 14–20 hours.

For imaging, the microdevice well was filled with ∼75 µl 0.7X Egg Salts (1X Egg Salts: 118 mM NaCl, 40 mM KCl, 3.4 mM MgCl2, 3.4 mM CaCl2, 5 mM HEPES pH 7.4), 1–3 worms were placed on the dissection board, cut open with a scalpel, and the one-cell embryos were swept towards the wells using an eyelash tool. The microdevice was placed on a metal slide with an opening at the location of the well, the slide was fastened to the microscope stage, and time-lapse imaging was initiated. When the embryos reached the desired stage, the medium in the well was exchanged by using a syringe with a blunt-end needle to remove the existing medium and adding fresh medium supplemented with the drug of interest with a pipette. Drugs used: 10 µg/ml nocodazole (Sigma, # M1404), 10 µM Latrunculin A (Sigma, # L5163), and 20 µM c-lactocystin-ß-lactone (Calbiochem, # 426102). At the end of each experiment, medium containing 33 µM FM4-64 (Molecular Probes, # T13320) was added to the well to confirm that the imaged embryo was permeable.

### Live imaging

Embryos were imaged at 21°C using a 60× 1.4 NA PlanApochromat lens on a spinning disk confocal setup mounted on a Nikon TE2000-E inverted microscope equipped with a solid-state laser combiner (ALC) – 491 nm and 561 nm lines – a Yokogawa CSU10 head and a CCD Clara camera (Andor Technology). Acquisition parameters, shutters, and focus were controlled by iQ 1.10.0 software (Andor Technology). Exposure times were 200 miliseconds for microtubules, DNA and plasma membrane GFP-labeled probes (laser power = 100 mW), and 400 miliseconds for the mCherry-labeled histone (laser power = 100 mW). Imaging conditions were 5×2 µm GFP/RFP z-series every 20 seconds. Analysis of acquired images was performed with MetaMorph software (Molecular Devices, Downington, PA).

### Microdevice fabrication

The microdevice (Fig. 3 and S3) consisted of a composite chip attached to a 24×60 mm #1.5 microscope coverslip. The composite chip was assembled from three parts: 1) a rectangular 14×16 mm, 3 mm thick polydimethylsiloxane (PDMS, Sylgard 184 by Dow Corning) chip with a rectangular 8×6.4 mm opening in the middle and a 6×10 mm, 250 µm deep counter-groove on its bottom; 2) a 6×10 mm, 200 µm thick plate micro-machined of a UV-curable epoxy that was inserted into the groove in the PDMS chip (dissection board); and 3) a 14×16 mm, 150 µm thick layer of PDMS with an array of 16 square 300×300 µm through-holes arranged in two rows, with a 700 µm distance between adjacent holes in a row and a 800 µm distance between the rows (array of microwells).

The 3 mm PDMS chips were cast using a master mold made of a resin (clear PolyJet FC720) on a high-resolution solid printer (PolyJet™ with an *x*-*y*-*z* resolution of 600-300-1600 dpi; Redeye, Eden Prairie, MN). A single cast made by baking PDMS on the mold in an 80°C oven for 90 min produced 12 individual chips. The high *z*-axis resolution of the mold (∼16 µm) was essential to generate counter-grooves with the depth closely matching the thickness of the epoxy plates (250 vs. 200 µm). To make the epoxy plates, a 5 inch silicon wafer was treated with trimethylchlorosilane (TMCS) to passivate its surface, a 200 layer of the UV-curable epoxy (SU8 2100 by MicroChem, Newton, MA) was spin-coated onto the wafer, pre-baked on a hot-plate, exposed to UV-light through a specially designed photomask, post-exposure baked, and developed. In the areas exposed to the UV light, cross-linked epoxy plates were formed. The plates were separated from the wafer and inserted into the grooves on the bottom surfaces of the PDMS chips.

To make the 150 µm thick perforated layer of PDMS, another 5 inch silicon wafer was spin-coated with SU8 2100 epoxy, which was exposed through another photo-mask and developed (with appropriate pre-baking and post-baking) to produce a relief with 12 separate arrays of 300 µm tall, 300×300 µm posts on the wafer surface. The wafer was then spin-coated with an ∼150 µm thick layer of PDMS pre-polymer, so that the top surfaces of the posts were exposed (PDMS free) [16]. The wafer was placed on a leveled plate in a 65°C oven for 7 min to let the pre-polymer reflow and partially cure. The PDMS chips with inserted epoxy plates were then manually aligned with respect to the arrays of posts on the wafer, and the wafer with the PDMS chips was baked in an 80°C oven to completely cure the 150 µm PDMS layer and permanently bond the chips to it. The PDMS layer was then cut around the edges of the 3 mm PDMS chips, and the composite chips with the epoxy plates and perforated PDMS membranes at the bottom were carefully separated from the wafer. To complete the microdevices, the bottom sides of the composite chips were reversibly bonded to coverslips.

In a complete microdevice, the 300×300 µm through-holes in the 150 µm PDMS layer formed microwells for immobilization of *C. elegans* embryos, the epoxy plate formed an integrated dissection board for adult *C. elegans* hermaphrodites, and the 8×6.4 mm opening in the 3 mm PDMS chip formed a macroscopic well for culture medium. To facilitate loading of medium into the microwells, microdevices were treated with air plasma (Plasma-Preen II by Plasmatic Systems Inc.) for 5 seconds to make the PDMS and glass surfaces hydrophilic.

### Medium exchange in the microwells

To test medium exchange, the medium in the microwells was exchanged by consecutive steps of aspiration from the 8×6.4 mm well with a 200 µl pipette and dispensing new medium into the well. During this process, the pipette tips never touched the microdevice, which limited the degree of medium exchange in a single aspiration/dispensing step. To estimate the time required for the small molecule concentration in the microwells to equilibrate with that in the well and to measure the eventual small molecule concentration in the microwells, we filled the well with a 60 ppm (by weight) solution of fluorescein (molecular weight ∼376 Da, coefficient of diffusion *D*≈4.5×10^−6^ cm^2^/s) in pH = 7.5 phosphate buffer and measured its fluorescence in the microwells with a video-microscopy setup consisting of an inverted fluorescence microscope (Nikon Diaphot) with a 60× 1.2 NA water immersion objective, 0.42× video coupler, and Sony XCD-X700 digital camera. The fluorescence illumination was derived from a 455 nm LED driven by a stabilized power supply. We used low illumination intensity in combination with a large exposure time (0.2 seconds) and binning over a 50×50 pixel region to minimize photobleaching. The microscope was focused ∼50 µm above the glass surface in one of the 300×300 µm microwells, corresponding to the approximate position of an embryo. Because of the high numerical aperture of the objective (1.2), the fluorescent signal was collected from a relatively thin layer around the focal plane, and the initial concentration of 60 ppm was sufficiently low to make the fluorescent signal proportional to the current fluorescein concentration as fluorescein was diluted from the microwell. Therefore, after subtraction of the background and normalization to the initial level, the fluorescence signal was representative of the fluorescein concentration normalized to its initial value (60 ppm solution).

Dependence of the normalized fluorescein concentration on time was recorded during 5 consecutive steps of aspiration of the medium from the well and dispensing plain buffer into the well (Fig. S3B). The time dependence showed that each dispensing step resulted in a rapid (∼2 s) drop in fluorescein concentration followed by a gradual (∼8 s) increase to a new level, which was significantly lower than the original concentration. The rapid drop was caused by convective flow through the microwell that propelled non-fluorescent buffer into the microwell. The convective flow was readily observable using tracer particles, but was too weak to move *C. elegans* embryos. The subsequent gradual increase of the concentration was likely due to diffusive mixing of the relatively concentrated fluorescein solution remaining in the well after the aspiration step. (The diffusion time *h* ^2^/(2*D*), with *h* = 150 µm, is ∼25 seconds and thus comparable with the time scale of the gradual concentration decrease). It is worth noting that since the medium exchange is mostly driven by convection rather than diffusion, the exchange time is not expected to be substantially greater for macromolecular compounds with moderate molecular weights.

Most importantly, after each of the medium exchange (aspiration/dispensing) steps, the concentration in the microwell rapidly decreased to ∼42% (on average) of its pre-exchange value. (The individual medium exchange steps are similar, when viewed in the semi-logarithmic coordinates, but not identical because the procedure is manual and thus not perfectly reproducible). So, the fluorescein concentration decreased to ∼3% of its initial value (97% exchange) after 4 aspiration/dispensing steps (∼55 seconds) and to 1.3% (98.7% exchange) after 5 steps (∼70 seconds). Therefore, the data in figure S3B indicates that in addition to drug addition the near complete removal of a substance from the medium in the well can also be achieved by repeated aspiration/dispensing within a ∼1–2 min time frame.

## Supporting Information

Figure S1 **Optimization of** ***perm-1*** **RNAi by soaking.** After generating a new batch of *perm-1* dsRNA or when using a new worm strain, soaking RNAi conditions were optimized using this protocol.
(EPS)

Figure S2 **Eggshell permeabilization using feeding RNAi.** **A.** Optimization of *perm-1* RNAi by feeding. When using a new worm strain, feeding RNAi conditions were optimized using this protocol. **B.** Graph showing the percent of embryos that were permeable (***green bars***) and that exhibited polar body resorption defects (***red bars***) after L4 or young adult worms were fed bacteria expressing *perm-1 dsRNA* for the indicated time intervals.
(EPS)

Figure S3 **The microdevice.** **A.** Schematic drawings of the top view and cross-sectional view of the microdevice as well as the microwells. **B.** Concentration of fluorescein (in semi-logarithmic scale) in a 300×300 µm microwell normalized to its initial concentration (60 ppm) as a function of time during a medium exchange procedure composed of 5 consecutive steps of aspiration of the existing medium from the 6×8 mm well and dispensing of plain buffer into the well (at time points of about 5, 21, 40, 58, and 72 seconds). The small molecule concentration in the microwells reaches equilibrium ∼5 s after medium replacement. A single cycle of aspiration/dispensing leads to an equilibrium small molecule concentration in the well that is ∼60% of that in the added buffer.
(EPS)

Movie S1 **Images of control and nocodazole-treated permeable embryos expressing GFP-histone H2B and GFP-alpha-tubulin.** A control embryo (***left***) and an embryo in which the media was replaced with media containing 10 µg/ml nocodazole just after nuclear envelope breakdown (***right***) are shown. Nocodazole addition results in rapid microtubule depolymerization and a decrease in the distance between the spindle poles. The chromosomes do not align in a metaphase plate and the embryo does not proceed into anaphase. Images are projections of 5 confocal sections, 2 µm apart, acquired every 20 seconds. Playback rate is 40× real time.
(MOV)

Movie S2 **Images of a permeable embryo expressing GFP-histone H2B and GFP-alpha-tubulin treated with nocodazole during pronuclear migration.** Addition of 10 µg/ml nocodazole prevents the pronuclei from moving towards each other but does not prevent chromosome condensation and nuclear envelope breakdown. Images are projections of 5 confocal sections, 2 µm apart, acquired every 20 seconds. Playback rate is 40× real time.
(MOV)

Movie S3 **Images of a permeable embryo expressing GFP-histone H2B and GFP-alpha-tubulin treated with nocodazole during anaphase.** Addition of 10 µg/ml nocodazole just after anaphase onset results in rapid microtubule depolymerization and failure of cytokinesis. Images are projections of 5 confocal sections, 2 µm apart, acquired every 20 seconds. Playback rate is 40× real time.
(MOV)

Movie S4 **Embryos co-expressing a GFP-labeled plasma membrane probe (** ***green*** **) and mCherry-labeled histone H2B (** ***red*** **) undergoing the first mitotic division in the absence (** ***left*** **) or in the presence of 10 µM Latrunculin A (** ***right*** **).** Addition of latrunculin A during anaphase results in failure of cytokinesis but does not block nuclear division. Latrunculin A addition also results in the accumulation of the plasma membrane probe in the cytoplasm (in the region of the spindle poles) upon DNA decondensation. Images are projections of 5 confocal sections, 2 µm apart, acquired every 20 seconds. Playback rate is 40× real time.
(MOV)

Movie S5 **Images of permeable embryos expressing GFP-histone H2B and GFP-alpha-tubulin undergoing the first mitotic division in the absence (** ***left*** **) or presence of a proteosome inhibitor, c-lactocystin-ß-lactone (** ***right*** **).** Addition of 20 µM c-lactocystin-ß-lactone early, during anaphase of meiosis II, leads to arrest at metaphase of the first mitotic division. Images are projections of 5 confocal sections, 2 µm apart, acquired every 20 seconds. The playback rate is 40× real time.
(MOV)

Movie S6 **Images of permeable embryos expressing GFP-histone H2B and GFP-alpha-tubulin undergoing the first and second mitotic divisions in the absence (** ***left*** **) or presence of a proteosome inhibitor, c-lactocystin-ß-lactone (** ***right*** **).** Addition of 20 µM c-lactocystin-ß-lactone before NEBD delayed the metaphase-anaphase transition but the embryo did not arrest until metaphase of the second mitotic division. Images are projections of 5 confocal sections, 2 µm apart, acquired every 20 seconds. Playback rate is 40× real time.
(MOV)

Spreadsheet S1 **Excel sheet summarizing the results of the eggshell permeability screen.**
(XLSX)

Table S1 **Strains used in this study.**
(DOCX)
