# Supplementary material for: Acute Drug Treatment in the Early C. elegans Embryo
Source: PLoS One. 2011 Sep 14;6(9):e24656. doi: 10.1371/journal.pone.0024656 (PMC3173474; doi:10.1371/journal.pone.0024656)
Supplement: Table S1 — Strains used in this study. (DOCX) [file pone.0024656.s011.docx]

**Table S1.** Strains used in this study

| **Strain** | **Genotype** |
| --- | --- |
| N2 | Ancestral |
| OD52 | unc-119(ed3) ruIs32[pAZ132; pie-1/GFP::histone H2B] III; ltIs24 [pAZ132; pie-1/GFP::tba-2; unc-119 (+)] |
| OD95 | unc-119(ed3) III; ltIs37 [pAA64; pie-1/mCHERRY::his-58; unc-119 (+)] IV; ltIs38 [pAA1; pie-1/GFP::PH(PLC1delta1); unc-119 (+)] |
